# Comparative developmental toxicity and oxidative stress of synthetic and natural yellow food colorants in zebrafish (*Danio rerio*) embryos

**DOI:** 10.1016/j.toxrep.2026.102343

**Published:** 2026-09-16

**Authors:** Mahdieh Kiannezhad, Jalal Hassan, Mohammad Kazem Koohi, Amir Moghaddam Jafari

**Affiliations:** a Department of Basic Sciences, Faculty of Veterinary Medicine, Ferdowsi University of Mashhad, Mashhad, Iran; b Department of Toxicology, School of Veterinary Medicine, University of Tehran, Tehran, Iran

**Keywords:** Tartrazine, Safflower, Saffron, Developmental toxicity, Oxidative stress, Zebrafish

## Abstract

Food colorants are widely used in the food industry; however, information regarding the comparative developmental toxicity of natural and synthetic yellow colorants remains limited. This study evaluated the embryotoxic, teratogenic, and oxidative stress effects of saffron (*Crocus sativus* L.), safflower (*Carthamus tinctorius* L.), and the synthetic azo dye tartrazine using zebrafish (*Danio rerio*) embryos. Embryos were exposed to serial concentrations (0.0001–1%, w/v) of each colorant from 2 to 96 h post-fertilization (hpf). Survival, hatchability, morphological abnormalities, median lethal concentration (LC₅₀), median effective concentration (EC₅₀), teratogenic index (TI), and malondialdehyde (MDA) levels were assessed. Safflower extract exhibited the greatest embryotoxicity, characterized by reduced survival, delayed hatching, elevated mortality, and the lowest LC₅₀ value among the tested colorants. Tartrazine produced pronounced, concentration-dependent developmental abnormalities, including cardiac edema, yolk sac edema, spinal deformities, and pigmentation abnormalities, and showed a comparatively higher teratogenic index than safflower, although both values remained below the classical TI > 1 threshold for teratogenicity. In contrast, saffron extract showed comparatively low toxicity, with minimal developmental abnormalities and higher survival rates at sublethal concentrations. Oxidative stress analysis revealed significant increases in MDA levels following exposure to safflower and tartrazine, indicating enhanced lipid peroxidation and oxidative damage. Principal component analysis further distinguished the toxicological profiles of the tested colorants, separating safflower-associated embryotoxicity from tartrazine-associated comparatively higher TI than safflower. These findings demonstrate that both natural and synthetic yellow food colorants can adversely affect embryonic development in a concentration-dependent manner and suggest that natural origin alone does not necessarily predict biological safety. The zebrafish embryo model provides a useful platform for comparative food colorant safety assessment and developmental toxicity screening.

## Highlights

• Zebrafish embryos were used to compare natural and synthetic yellow colorants.
• Safflower extract exhibited the highest embryotoxicity and hatch inhibition.
• Tartrazine exhibited the greatest severity of morphological abnormalities and developmental defects.
• Oxidative stress was associated with developmental toxicity in zebrafish.
• Natural food colorants were not universally safer than synthetic additives.

## Graphical Abstract

**Figure:**
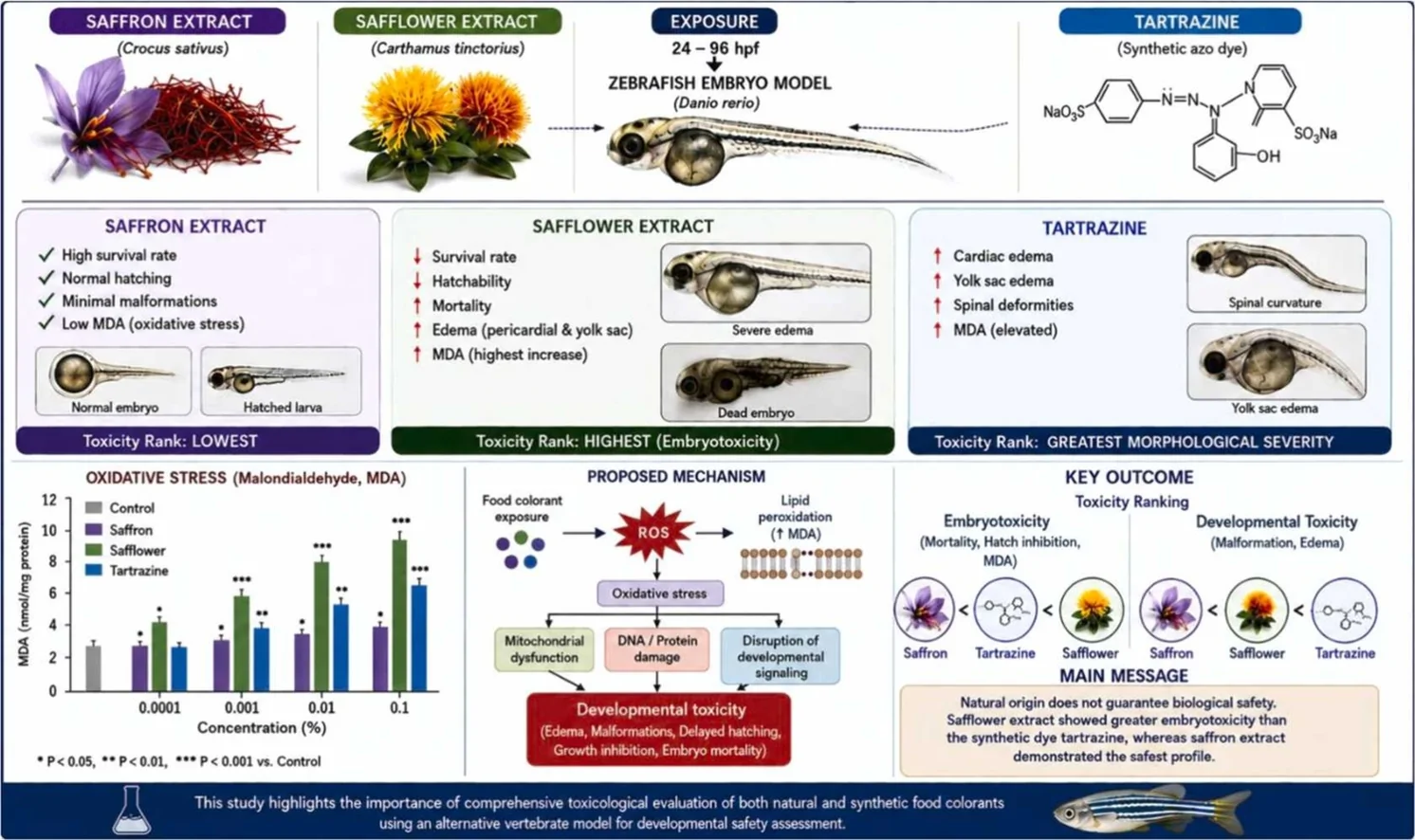

## 1 Introduction

Food colorants are extensively used in the food, beverage, pharmaceutical, and cosmetic industries to enhance visual appearance, improve consumer acceptance, and maintain product consistency. Both synthetic and natural pigments are commonly incorporated into confectionery products, dairy products, beverages, processed foods, and medicinal formulations. The increasing consumption of colored food products has raised concerns regarding the potential biological and toxicological effects of food additives, particularly during early developmental stages when organisms are highly sensitive to chemical exposure [1,2].

**Fig. 1:**
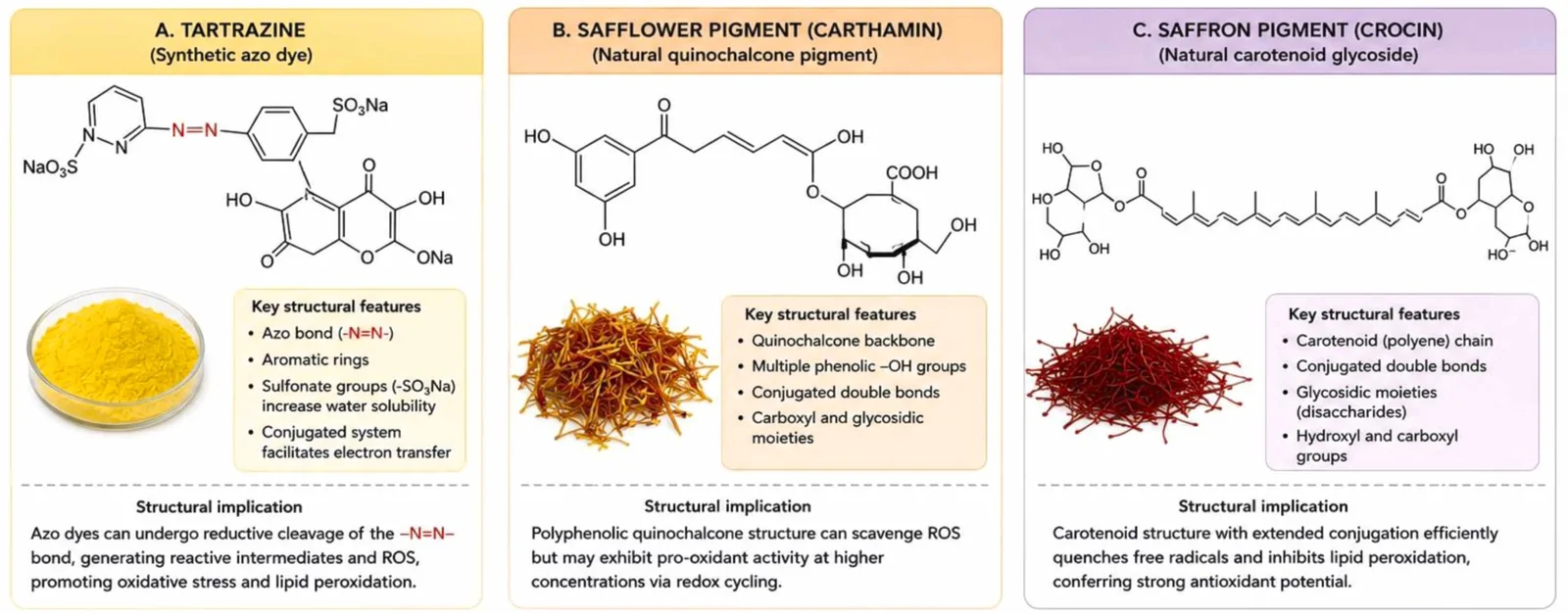
Representative chemical structures and key structural features of the tested yellow food colorants.

Among synthetic food colorants, tartrazine (TTZ; E102) is one of the most widely utilized yellow azo dyes because of its high stability with melting point of 300 °C, strong coloring capacity, water solubility, and low production cost. Structurally, tartrazine contains aromatic rings linked by azo (-N = N-) bonds, which are known to undergo metabolic reduction and generate reactive intermediates and free radicals (Fig. 1A) [3,4]. Previous experimental studies have associated tartrazine exposure with oxidative stress, neurotoxicity, hepatotoxicity, behavioral alterations, allergic reactions, hyperactivity, and developmental abnormalities [[5], [6], [7]]. The pro-oxidant activity of azo dyes may disrupt cellular redox homeostasis and induce excessive formation of reactive oxygen species (ROS), leading to lipid peroxidation, mitochondrial dysfunction, protein oxidation, inflammation, and DNA damage [8,9]. Oxidative stress is considered one of the principal mechanisms underlying chemically induced developmental toxicity. During embryogenesis, rapidly proliferating tissues possess limited antioxidant defense capacity, rendering embryos particularly susceptible to ROS-mediated cellular injury [10,11]. Lipid peroxidation represents a major consequence of oxidative stress and contributes to disruption of membrane integrity and cellular function. Malondialdehyde (MDA), a stable end-product of lipid peroxidation, is widely recognized as a sensitive biomarker of oxidative membrane damage and oxidative stress in toxicological investigations [12,13]. Therefore, assessment of MDA levels may provide important insight into the oxidative effects of synthetic and natural food colorants during embryonic development.

In recent years, increasing scientific and industrial interest has focused on natural pigments as potential alternatives to synthetic food dyes due to growing consumer demand for naturally derived additives [14]. Safflower (*Carthamus tinctorius* L.) and saffron (*Crocus sativus* L.) are natural yellow colorants rich in biologically active phytochemicals with antioxidant properties [15,16]. Safflower contains flavonoids, quinochalcone compounds, and polyphenolic constituents, whereas saffron is characterized by the presence of crocin, crocetin, picrocrocin, and safranal (Fig. 1B,C) [17,18]. These compounds possess conjugated double-bond systems and hydroxyl-containing functional groups capable of scavenging free radicals and reducing oxidative injury [19,20]. In contrast to synthetic azo dyes, the chemical structures of safflower and saffron pigments are generally associated with antioxidant and cytoprotective activities [[21], [22], [23]]. Nevertheless, despite their medicinal importance and widespread use, the developmental toxicological profiles of these natural pigments remain insufficiently characterized. Importantly, natural origin does not necessarily guarantee biological safety, especially at high concentrations or prolonged exposure conditions.

The zebrafish (*Danio rerio*) embryo model has become an important vertebrate system for toxicological and developmental studies because of its genetic similarity to humans, external fertilization, rapid embryonic development, optical transparency, and high sensitivity to environmental toxicants [24,25]. Zebrafish embryos allow direct observation of developmental processes, including hatching, heartbeat, body morphology, survival, organogenesis, and behavioral responses [26,27]. Furthermore, the zebrafish model provides a cost-effective, reproducible, and ethically advantageous platform for evaluating oxidative stress and developmental toxicity induced by food additives and environmental chemicals [28,29].

Although several studies have separately investigated synthetic food dyes or the pharmacological properties of natural pigments, comparative studies evaluating synthetic and natural yellow food colorants using integrated developmental and oxidative stress biomarkers remain limited. Moreover, little information is available regarding the structure–activity relationships underlying differences in the toxicological behavior of tartrazine, safflower extract, and saffron extract. Comparative assessment of these pigments may provide valuable information regarding the relative safety of synthetic and natural food colorants and their potential mechanisms of toxicity [[30], [31], [32]].

Therefore, the present study aimed to comparatively evaluate the developmental toxicity and oxidative stress-inducing effects of tartrazine, safflower (*Carthamus tinctorius* L.) extract, and saffron (*Crocus sativus* L.) extract in zebrafish larvae using morphological assessment and malondialdehyde (MDA) analysis. It was hypothesized that tartrazine would induce greater oxidative stress and developmental toxicity than safflower and saffron extracts due to its azo dye structure and higher pro-oxidant potential.

## 2 Materials and methods

### 2.1 Preparation of test substances

Tartrazine powder (FD&C Yellow No. 5, E102, CAS 1934–21–0; Bin Afif Co., Saudi Factory for Food Colors and Improvers, Jeddah, Saudi Arabia) was used as a commercial food-grade colorant; the manufacturer did not provide a certified purity or analytical grade, though commercial tartrazine typically meets a minimum assay of 85% total colouring matter per JECFA/EU specifications. Due to its high-water solubility, the powder was dissolved in 10 mL of distilled water to prepare a 10% (w/v) aqueous stock solution. Serial dilutions of the stock solution were subsequently prepared to obtain final exposure concentrations of 1%, 0.1%, 0.01%, 0.001%, and 0.0001% for zebrafish embryotoxicity assays.

Dried safflower petals (*Carthamus tinctorius* L.) and dried saffron stigmas (*Crocus sativus* L.) were separately ground into fine powders. Commercial Iranian saffron (*Crocus sativus* L.), labeled as "All Red" and first-grade filamentous saffron, was obtained from Mostafavi Saffron (Iran, Mashhad), with product batch/lot number 3010128108056 and registration NO: 62388. Both materials were commercial food-grade products; the supplier did not assign a specific certified analytical or pharmacopoeial grade, and no additional grade-specific certification was obtained for this study. For each extract, 1 g of powdered material was mixed with 10 mL of distilled water and agitated on an orbital shaker at 100 rpm (approximately 1.9 × g) for 10 min. Freshly boiled distilled water (≈100 °C) was used for safflower extraction, whereas room-temperature distilled water was used for saffron extraction, mirroring typical culinary preparation practices for each plant material: safflower petals are traditionally steeped in boiling water to release the water-soluble pigments, whereas saffron stigmas are conventionally infused in cool-to-room-temperature water to preserve heat-labile constituents such as crocin and safranal. Both mixtures were subsequently subjected to an identical hydrothermal extraction step in a bain-marie at 90–95 °C for 30–40 min to facilitate the release of pigments and bioactive constituents; the differing initial mixing temperature therefore represents the only temperature-related variable distinguishing the two extraction procedures prior to this common heating step. Following extraction, the suspensions were sequentially filtered through a mesh strainer and standard filter paper to remove insoluble particles and plant debris. The filtrates were subsequently centrifuged at 700 × g for 6 min to obtain clear aqueous extracts. The collected supernatants were considered 10% (w/v) stock extracts. Serial dilutions were then prepared from each stock solution to yield final test concentrations of 1%, 0.1%, 0.01%, 0.001%, and 0.0001% for zebrafish embryo exposure experiments.

### 2.2 Spectrophotometric analysis of tested colorants

The absorbance characteristics of tartrazine, safflower extract, and saffron extract were analyzed using a UV–Visible spectrophotometer to evaluate their optical properties and pigment profiles. Stock solutions (10%, w/v) of each colorant were diluted appropriately with distilled water prior to analysis. Spectral measurements were performed within the wavelength range of 200–700 nm using quartz cuvettes with a 1-cm optical path length, while distilled water was used as the blank reference.

The maximum absorption wavelength (λ max) of each tested colorant was determined from the obtained absorption spectra. Spectrophotometric analysis was conducted to compare the optical behavior and pigment-related characteristics of synthetic and natural yellow food colorants prior to zebrafish exposure experiments.

### 2.3 Maintenance and collection of adult zebrafish

Seventy-nine adult zebrafish (*Danio rerio*), approximately 5 months old and measuring 1–2 cm in length, were obtained from the Zebrafish Laboratory, Department of Toxicology, Faculty of Veterinary Medicine, University of Tehran, Iran. Prior to experimentation, fish were acclimatized for nine days in aerated and conditioned system water under standardized laboratory conditions consistent with OECD Test Guideline 203. The stock population used for breeding and for the preliminary adult range-finding test (described below) consisted of 40 males and 39 females; these adults were not part of the embryo treatment groups reported in the Results. Zebrafish were maintained at 27.5 ± 0.5 °C under a controlled photoperiod of 14 h’ light and 10 h dark. Water quality parameters were carefully monitored throughout the acclimation and exposure periods to ensure optimal husbandry conditions. The physicochemical characteristics of the system water included a pH of 7.0 ± 0.2, dissolved oxygen levels above 6 mg/L, total hardness of 100–150 mg/L as CaCO₃, conductivity of 300–500 µS/cm, and ammonia concentrations below detectable toxic limits. Continuous aeration and partial water renewal were performed regularly to maintain stable environmental conditions and minimize stress-related variability [33,34]. As a preliminary, non-definitive range-finding step, and prior to designing the embryo concentration series described below, groups of seven adult zebrafish were transferred into individual 1-L glass tanks containing freshly prepared test solutions. In accordance with standardized acute toxicity protocols, fish were not fed during the 96-h exposure period. Animals were observed twice daily throughout the experiment for mortality and behavioral or physiological abnormalities. Dead fish were removed immediately upon detection. Mortality was defined by the absence of opercular movement and lack of response to gentle mechanical stimulation of the caudal fin. Clinical manifestations of toxicity, including abnormal swimming behavior, loss of equilibrium, respiratory distress, hypo activity, and alterations in skin or ocular pigmentation, were systematically recorded during the exposure period. This adult range-finding test was used solely to establish a workable upper concentration boundary for the subsequent embryo exposure experiments; it was not a standalone reportable endpoint, and adult fish were not otherwise included in the treatment groups described in the Results, which are based exclusively on the embryo exposures detailed in the following sections.

### 2.4 Zebrafish embryotoxicity assay

The zebrafish embryotoxicity assay was performed in accordance with OECD Test Guideline 236 for Fish Embryo Acute Toxicity (FET) testing. Adult male and female zebrafish were placed in breeding tanks at a ratio of 2:1 and maintained overnight under standard laboratory conditions. Each spawning tank was fitted with a plastic mesh breeding insert (3-mm pore size) that allowed fertilized eggs to settle at the bottom while preventing predation by adult fish [35,36].

Spawning was induced during the early light phase, approximately 12 h after the onset of darkness. Fertilized eggs were collected within 30–60 min after removal of the tank divider and rinsed twice with E3 embryo medium to eliminate debris and unfertilized eggs. The E3 medium consisted of 5 mM NaCl, 0.17 mM KCl, 0.33 mM CaCl₂·2H₂O, and 0.33 mM MgCl₂·7H₂O. Collected embryos were examined under an inverted microscope, and only normally developing and viable embryos were selected for subsequent experiments.

Selected embryos were individually transferred into pre-saturated 96-well microplates containing one mL E3 medium, with 60 embryos allocated to each treatment concentration (20 embryos per replicate, with three independent replicates per concentration). Embryos were exposed within 2 h post-fertilization (blastula stage) to different concentrations of the tested colorants (0, 0.0001, 0.001, 0.01, 0.1 and 1% w/v). Exposure plates were maintained at 28 ± 0.5 °C under controlled laboratory conditions, and the treatment solutions were renewed every 24 h to maintain chemical stability and water quality.

Embryonic mortality, larval survival, hatching success, and developmental abnormalities were assessed at 24-h intervals (24, 48, 72, and 96 hpf) over the 96-h exposure period using an inverted microscope (Zeiss, Germany; ×80 magnification). Hatching rate was calculated as the percentage of hatched embryos relative to the total number of viable embryos in each treatment group. Teratogenic endpoints, including yolk sac edema, spinal curvature, pericardial edema, developmental delay, and tail malformations, were systematically recorded throughout the study period.

All experimental procedures involving zebrafish were conducted in accordance with institutional ethical guidelines and were approved by the Research Ethics Committee of Ferdowsi University of Mashhad, Iran (Approval ID: IR.UM.REC.1402.055).

### 2.5 Experimental design and endpoint monitoring

As detailed above, viable zebrafish embryos were randomly distributed into sterile 96-well culture plates using the same exposure setup. Experimental groups included embryos exposed to tartrazine, safflower extract, and saffron extract at concentrations of 0 (control), 0.0001, 0.001, 0.01, 0.1 and 1% (w/v). Each concentration was tested in triplicate wells, and the entire experiment was independently repeated three times to ensure reproducibility and minimize inter-experimental variability.

Test concentrations were selected based on OECD recommendations (2013) and previously published zebrafish embryotoxicity protocols [37]. Exposure media were renewed every 24 h to maintain solution stability and appropriate water quality throughout the experimental period.

Embryonic survival, hatching success, and developmental toxicity were monitored daily using an inverted microscope. Acute toxicity and embryo lethality were evaluated at 96 hpf according to established apical endpoints of zebrafish embryo toxicity. Recorded abnormalities included embryo coagulation, absence of tail detachment, impaired somite formation, pericardial edema, yolk sac edema, spinal curvature, and tail malformations. The frequency of each developmental endpoint was calculated as the percentage of affected embryos relative to the total number of surviving embryos within each treatment group.

### 2.6 Morphological scoring

Morphological abnormalities in each anatomical region were documented photographically and quantitatively scored on a six-level severity scale (0.5–5, where 5 indicates normal morphology and 0.5 indicates near-complete structural loss), adapted from the dechorionated zebrafish teratogenicity scoring guidelines of Panzica-Kelly et al. [38] Scoring was performed independently for the following anatomical regions: tail (fin-fold integrity and notochord straightness), fins (pectoral fin formation and symmetry), heart (chamber looping and pericardial contour), somites (segmentation regularity and boundary definition), pectoral fins (presence, size, and symmetry), and facial region (jaw/mandible formation, eye size, and inter-ocular distance). Two independent, blinded observers scored a subset of embryos to verify scoring consistency.

### 2.7 Determination of LC₅₀, EC₅₀, and Teratogenic Index

The median lethal concentration (LC₅₀) at 96 hpf was defined as the concentration causing mortality in 50% of exposed embryos. The median effective concentration (EC₅₀) was defined as the concentration inducing developmental abnormalities in 50% of surviving embryos. Both LC₅₀ and EC₅₀ values were estimated using probit regression analysis.

The teratogenic index (TI) was calculated as the ratio of LC₅₀ to EC₅₀ (TI = LC₅₀/EC₅₀) to describe the relative separation between the concentrations associated with lethality and developmental effects. A TI value > 1 is conventionally interpreted as indicating that developmental effects occur at concentrations below those associated with lethality. In the present study, both calculated TI values were below 1; therefore, the TI values were interpreted as comparative measures of the relationship between lethal and effect-associated concentrations rather than as evidence of classical teratogenicity.

### 2.8 Lipid Peroxidation Assay (Malondialdehyde Determination)

Lipid peroxidation levels in zebrafish embryos exposed to the tested colorants were evaluated by measuring malondialdehyde (MDA) content using the thiobarbituric acid reactive substances (TBARS) assay. Following completion of the exposure period, pooled zebrafish embryos from each experimental group were collected, rinsed with cold phosphate-buffered saline (PBS, pH 7.4), and homogenized in ice-cold PBS using a glass homogenizer. The homogenates were centrifuged at 3000 × g for 10 min at 4 °C, and the resulting supernatants were used for biochemical analysis.

For MDA determination, 0.5 mL of the supernatant was mixed with 1 mL of thiobarbituric acid (TBA) reagent containing trichloroacetic acid (TCA) and thiobarbituric acid under acidic conditions. The reaction mixtures were heated in a boiling water bath at 95–100 °C for 30 min to allow formation of the MDA–TBA adduct. After cooling to room temperature, samples were centrifuged at 3000 × g for 10 min to remove precipitated material.

The absorbance of the supernatants was measured spectrophotometrically at 532 nm using a UV–Visible spectrophotometer. MDA concentrations were calculated using the molar extinction coefficient of the MDA–TBA complex and expressed as nmol MDA/mg protein. Protein concentrations of the samples were determined separately using the to normalize oxidative stress measurements, which are expressed as nmol MDA/mg protein. For each replicate, embryos were pooled in groups of [author: insert number of embryos pooled per replicate] prior to homogenization. The assay was performed in triplicate for each treatment group to ensure analytical reliability and reproducibility. Increased MDA levels were interpreted as indicators of enhanced lipid peroxidation and oxidative stress induced by exposure to the tested food colorants.

### 2.9 Statistical Analysis

All experiments were conducted in triplicate, and data are presented as mean ± standard deviation (SD). Statistical analyses were performed using SPSS software (version 27.0; IBM Corp., Armonk, NY, USA) and GraphPad Prism (version 8.0). The effects of colorant type and concentration on embryonic mortality, hatching rate, teratogenicity endpoints, and malondialdehyde (MDA) levels were analyzed using two-way analysis of variance (ANOVA), followed by Tukey’s post hoc multiple comparison test. Differences were considered statistically significant at *p* < 0.05.

Prior to all analyses, data normality and homogeneity of variance were assessed using the Shapiro–Wilk and Levene’s tests, respectively.

LC₅₀ and EC₅₀ values at 96 hpf (as defined above) were estimated using probit regression analysis in SPSS, with confirmation by GraphPad Prism and, where applicable, PoloPlus software to calculate 95% confidence intervals. PoloPlus was used preferentially for confidence interval estimation because it is purpose-built for concentration–response (probit) analysis in ecotoxicology and directly outputs fiducial 95% confidence limits alongside goodness-of-fit diagnostics, which require additional manual steps to obtain in general-purpose statistical software [[39], [40], [41]].

### 2.10 Probit-based estimation of LC₅₀ and EC₅₀

For LC₅₀ determination, mortality data were entered into SPSS according to the following structure:

• Covariate: tested concentration (%)
• Total exposed embryos: 20 embryos per concentration per replicate
• Response frequency: number of dead embryos recorded at each concentration

For EC₅₀ determination, the same probit structure was used, with response frequency defined as the number of embryos exhibiting developmental abnormalities at each concentration. In both models, concentration was log₁₀-transformed prior to fitting a probit link function.

LC₅₀ values and corresponding 95% confidence intervals were obtained from the confidence limits output table at a response probability of 0.50. EC₅₀ values were calculated using the same probit regression approach based on the frequency of morphological abnormalities, including spinal deformities, yolk sac edema, pericardial edema, developmental delay, and tail malformations. The concentration corresponding to a 50% probability of developmental toxicity was considered the EC₅₀ value.

Principal component analysis (PCA) was performed to investigate relationships among toxicological and developmental endpoints and to identify patterns of similarity among the tested food colorants. The variables included mortality rate, hatchability, incidence of edema, malformation score, teratogenic index (TI), and malondialdehyde (MDA) levels measured at 96 hpf. Prior to analysis, data were standardized using z-score normalization to eliminate differences in measurement scales, and the suitability of the dataset for PCA was confirmed using the Kaiser–Meyer–Olkin (KMO) measure of sampling adequacy and Bartlett’s test of sphericity. Varimax (orthogonal) rotation was applied to improve the interpretability of component loadings. The first two principal components (PC1 and PC2) were extracted and used to visualize clustering patterns and variable contributions among treatment groups. PCA was conducted using OriginPro (OriginLab Corporation, Northampton, MA, USA).

## 3 Results

### 3.1 Spectrophotometric analysis findings

**Fig. 2:**
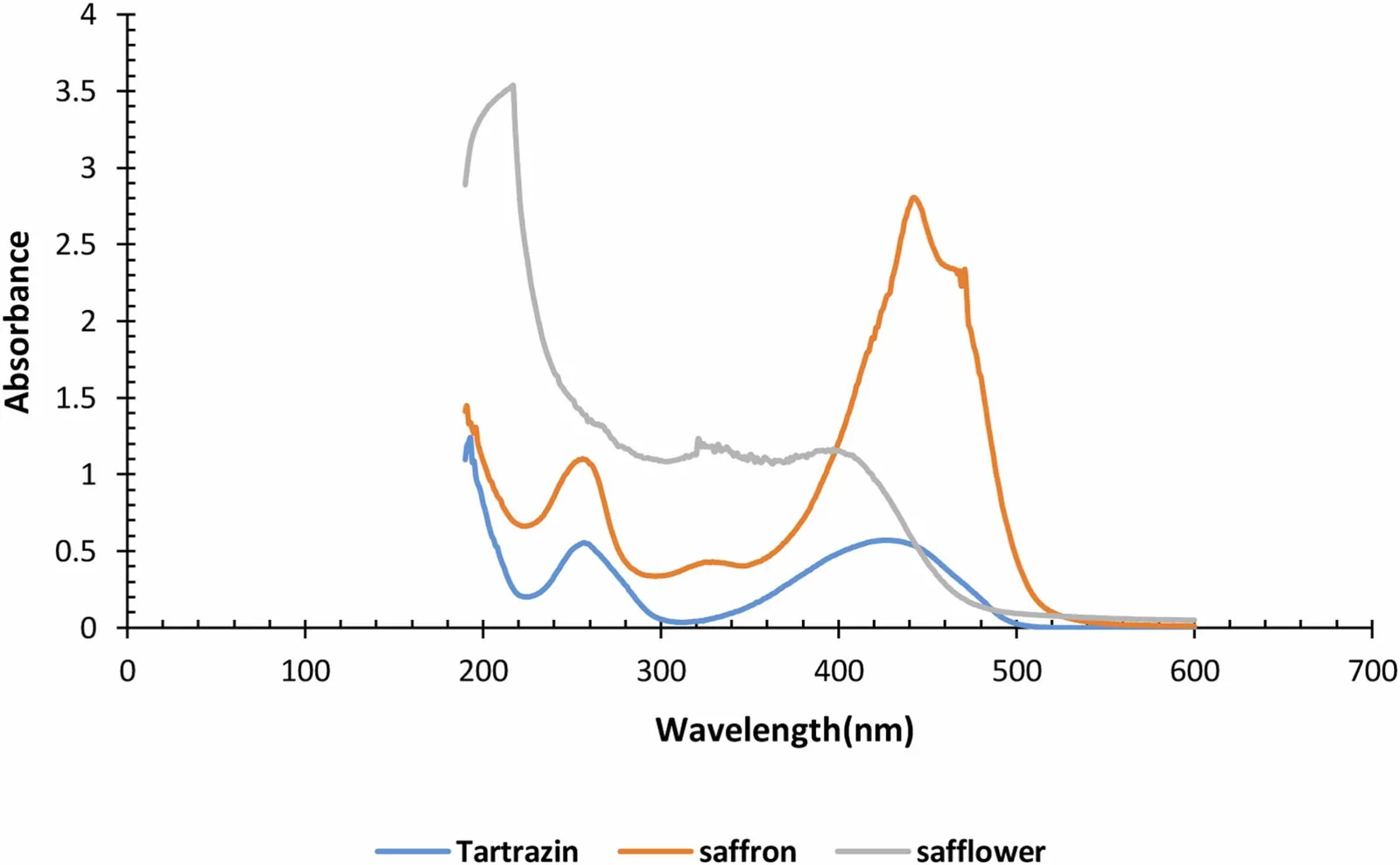
UV–Visible absorption spectra of tartrazine, safflower extract (*Carthamus tinctorius* L.), and saffron extract (*Crocus sativus* L.) measured between 200 and 700 nm. Tartrazine showed a distinct absorption maximum (240 nm) characteristic of azo dyes in the visible region, while safflower and saffron extracts exhibited broader absorption bands associated with natural carotenoid and flavonoid pigments (200–500 nm). Spectrophotometric characterization was performed to compare the pigment profiles and optical properties of natural and synthetic yellow food colorants used in the developmental toxicity assays.

The UV–Visible absorption spectra of saffron extract, safflower extract, and tartrazine are presented in Fig. 2. Distinct absorption profiles were observed among the tested colorants, reflecting differences in their pigment composition and chemical structure. Tartrazine exhibited a sharp and well-defined absorption peak within the visible region characteristic of synthetic azo dyes. In contrast, saffron and safflower extracts displayed broader absorption bands extending across the visible spectrum, consistent with the presence of multiple naturally occurring pigment constituents. Among the natural colorants, saffron demonstrated a prominent absorption peak corresponding to carotenoid-derived compounds, whereas safflower exhibited a broader spectral profile indicative of a complex mixture of flavonoid and quinochalcone pigments. The absorbance intensity of the tested colorants increased proportionally with concentration, confirming concentration-dependent optical behavior. The distinct spectral characteristics observed among the three colorants indicate substantial differences in pigment composition and molecular structure. These differences may contribute to the variations in developmental toxicity, morphological abnormalities, and oxidative stress responses observed in zebrafish embryos following exposure to the tested colorants.

### 3.2 Effects of TTZ, safflower and saffron extracts on zebrafish embryo viability

**Fig. 3:**
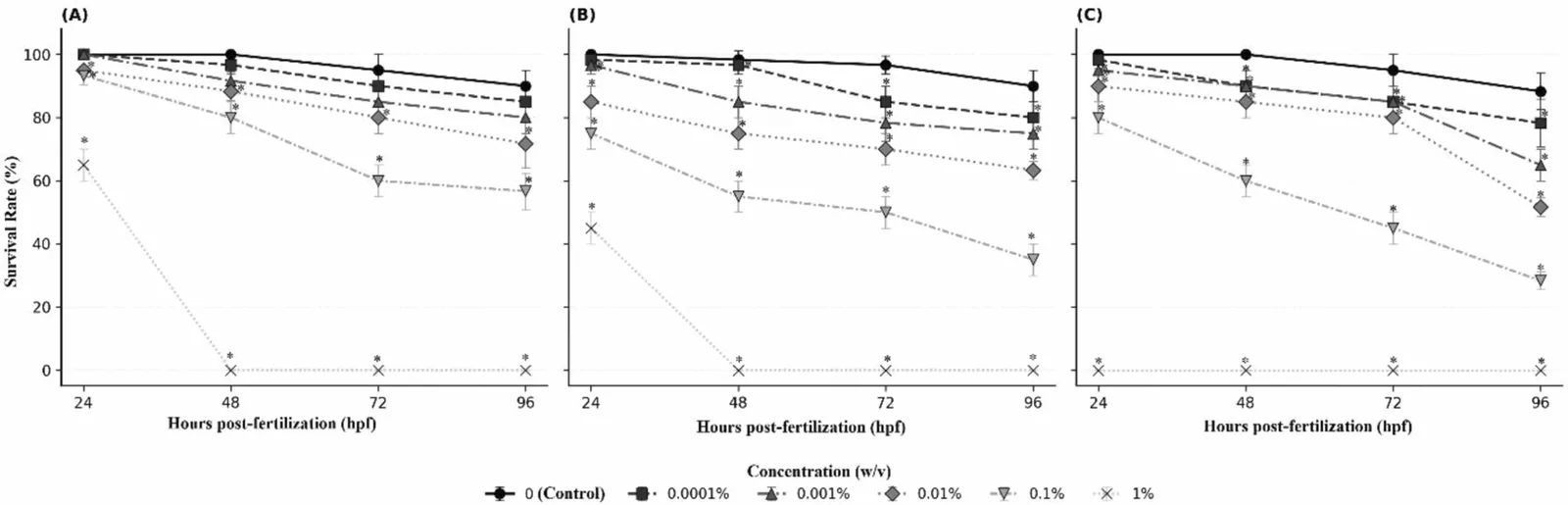
Survival rate (%) of zebrafish (*Danio rerio*) embryos following exposure to increasing concentrations of saffron extract (A), safflower extract (B), and tartrazine (C) during the developmental period from 24 to 96 h post-fertilization (hpf). Survival was monitored at defined time intervals throughout the exposure period. Data are presented as Mean ± SD for each experimental group. A concentration- and time-dependent reduction in embryo survival was observed, with safflower extract exhibiting the greatest embryotoxic effect among the tested colorants. Asterisks (*) indicate statistically significant differences compared with the untreated control group (P < 0.05).

The survival rate of zebrafish embryos exposed to saffron, safflower, and tartrazine across concentrations ranging from 0% to 1% was evaluated from 24 to 96 hpf (Fig. 3). The survival rate of zebrafish embryos decreased in a concentration- and time-dependent manner following exposure to saffron, safflower, and tartrazine from 24 to 96 hpf. At low concentrations (0.0001–0.001%), all three substances produced survival rates comparable to the untreated control throughout the exposure period. In contrast, moderate concentrations (≥0.01%) led to progressively reduced survival, becoming more pronounced at later time points. The highest concentration tested (1%) consistently induced rapid and complete mortality, typically by 24–48 hpf. At the 1% concentration, eggs exposed to safflower and saffron coagulated within 48 h, whereas eggs exposed to tartrazine coagulated more rapidly, within 24 h, indicating that tartrazine exerts faster acute toxicity at high concentrations while the natural extracts act over a comparatively longer, sub-acute exposure window. Among the tested agents, tartrazine exhibited the strongest acute toxicity, followed by safflower and saffron. Significant differences (*P* < 0.05) were observed primarily at concentrations ≥ 0.01% for all compounds across the experimental time course. Overall, the findings indicate concentration-dependent embryotoxicity with cumulative time-related effects, with tartrazine demonstrating the highest potency.

### 3.3 Effects of TTZ, safflower and saffron extracts on zebrafish egg hatchability

**Fig. 4:**
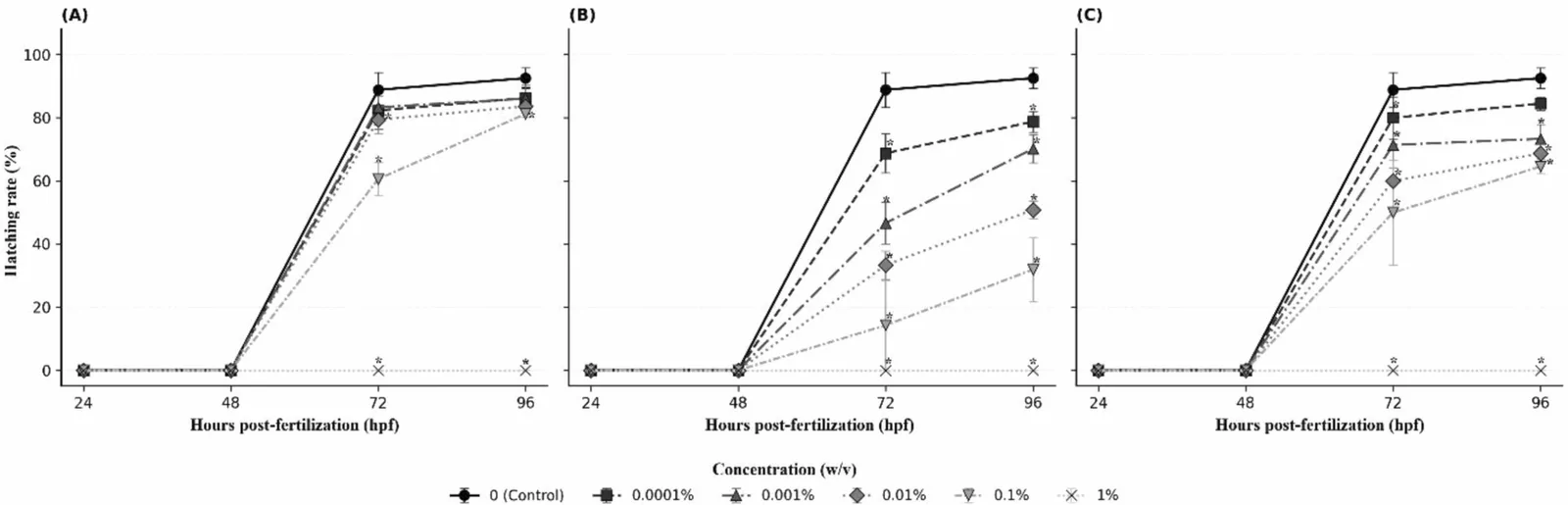
Hatching rate (%) of zebrafish *(Danio rerio)* embryos following exposure to increasing concentrations of saffron extract (A), safflower extract (B), and tartrazine (C) from 24 to 96 h post-fertilization (hpf). Hatching success was evaluated at designated developmental intervals throughout the exposure period. Data are expressed as Mean ± SD for each treatment group. Exposure to the tested colorants resulted in concentration-dependent alterations in embryo hatchability, with safflower extract producing the most pronounced inhibitory effect on hatching rate. Asterisks (*) indicate statistically significant differences compared with the untreated control group (P < 0.05).

The hatchability of zebrafish embryos exposed to saffron, safflower, and tartrazine from 24 to 96 hpf exhibited clear concentration-dependent differences (Fig. 4). No hatching occurred at 24 and 48 hpf across all treatment groups, consistent with normal developmental timing. At 72 hpf, embryos in the control and low-concentration groups (0.0001–0.001%) showed high hatchability rates, typically exceeding 80–90% for all three tested substances. However, moderate concentrations (0.01–0.1%) produced reduced hatchability, with the magnitude of impairment varying among treatments. The highest concentration tested (1%) severely suppressed hatching, with complete inhibition consistently observed at both 72 and 96 hpf. At 0.1% concentration of safflower (96 hpf) 60% of the eggs had not hatched, while in saffron, more than 85% had hatched. Among the three tested agents, safflower produced the most pronounced inhibition of hatching at moderate concentrations, followed by tartrazine and saffron. Statistically significant differences (*P* < 0.05) were observed primarily at concentrations ≥ 0.01% for both time points with active hatching (72–96 hpf). Overall, hatchability outcomes indicate that all three compounds exert dose-dependent embryotoxic effects, with tartrazine demonstrating the greatest potency in disrupting normal hatching.

### 3.4 Effects of TTZ, Safflower and Saffron Extracts on edema-related developmental toxicity

**Fig. 5:**
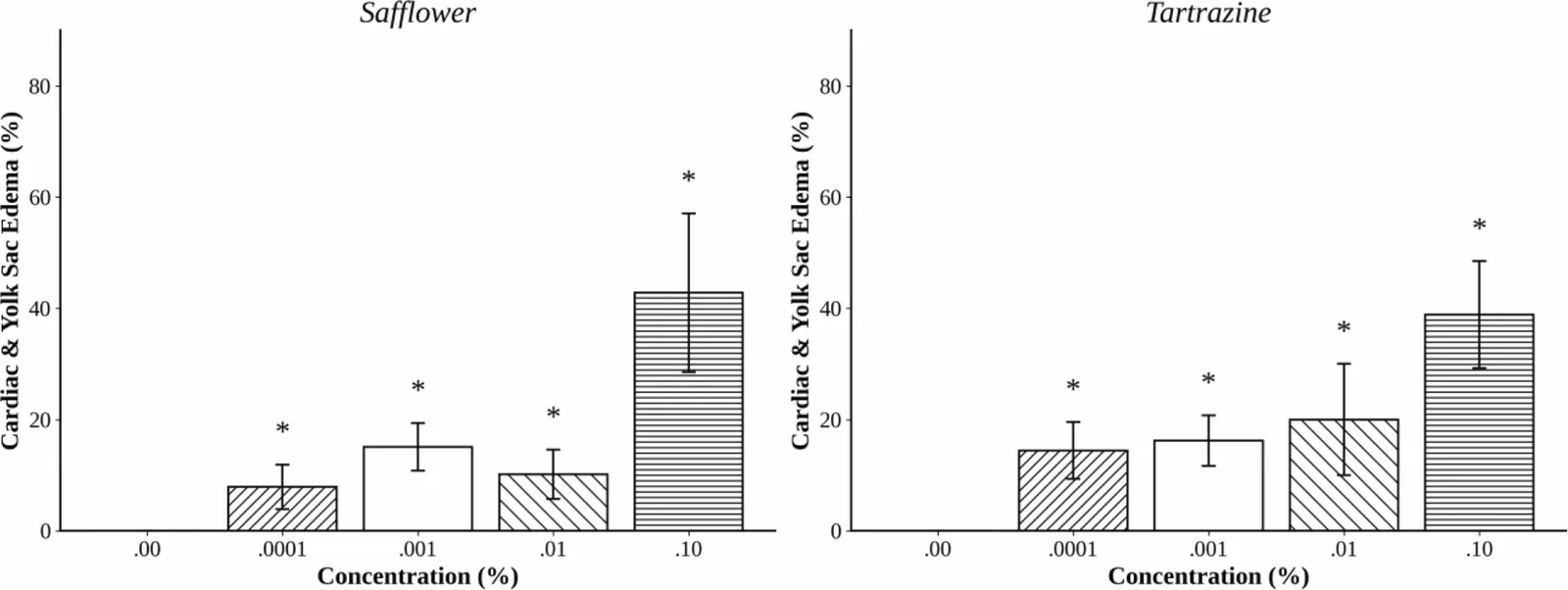
Incidence of cardiac and yolk sac edema in zebrafish (*Danio rerio*) embryos following exposure to increasing concentrations of safflower extract (left panels) and tartrazine (right panels). Morphological abnormalities were assessed during embryonic development up to 96 h post-fertilization (hpf). Data are presented as Mean ± SD for each treatment group. Both safflower extract and tartrazine induced concentration-dependent increases in edema formation, whereas no detectable morphological abnormalities, including cardiac or yolk sac edema, were observed in embryos exposed to saffron extract at any tested concentration. Asterisks (*) indicate statistically significant differences compared with the untreated control group (P < 0.05).

Cardiac and yolk sac edema were assessed at 96 hpf following embryonic exposure to safflower extract and the azo dye tartrazine (Fig. 5). Both compounds induced concentration-dependent morphological abnormalities, with increasing severity at higher exposure levels. In embryos exposed to safflower, edema incidence rose progressively at concentrations ≥ 0.0001%, with markedly elevated frequencies observed at 0.01% and a substantial peak at 0.1%, where edema affected more than half of the embryos. Tartrazine elicited a similar but more pronounced pattern, with significantly higher edema rates recorded even at low concentrations (0.0001–0.001%) and further exacerbated at 0.01% and 0.1%. Statistical significance was detected across most concentrations for both substances (*P* < 0.05). In contrast, embryos exposed to saffron exhibited no detectable cardiac or yolk sac edema at any tested concentration, indicating an absence of observable morphological toxicity within the experimental range. Overall, the findings reveal that tartrazine is the associated with the greatest severity of edema, followed by safflower, whereas saffron appears did not induce detectable cardiac or yolk sac edema conditions.

### 3.5 Effects of TTZ, safflower and saffron extracts on LC₅₀, EC₅₀, and teratogenic index-based evaluation of embryotoxicity

Quantitative toxicity assessment at 96 hpf revealed distinct differences in lethality and developmental toxicity among the three tested food dyes (Table 1). Based on percentage concentrations, safflower exhibited the greatest acute toxicity, with the lowest LC₅₀ value (0.004%), followed by tartrazine (0.009%) and saffron (0.017%). Hatching impairment occurred at higher concentrations than those affecting lethality. The EC₅₀ values for hatching showed that saffron (0.487%) required the highest concentration to elicit a 50% reduction followed by tartrazine (0.0354%) and safflower (0.031%), suggesting that hatching was more resilient to saffron exposure compared to the other two substances. Developmental abnormalities—specifically cardiac and yolk sac edema—were observed exclusively in embryos exposed to tartrazine and safflower. The EC₅₀ for edema was lower for safflower (0.127%) than for tartrazine (0.153%), while saffron did not induce detectable edema, and therefore no EC₅₀ or TI values were derived for this endpoint. The teratogenic index (TI), calculated as LC₅₀/EC₅₀ for edema, was used to describe the relative separation between lethal and effect-associated concentrations. Tartrazine exhibited a TI of 0.058, whereas safflower showed a TI of 0.031; both values fall below the classical TI > 1 threshold, indicating that lethality occurred at or below the concentrations producing malformation for both substances. The comparatively higher TI of tartrazine indicates a narrower separation between the concentrations associated with developmental effects and lethality relative to safflower, while remaining below the classical TI > 1 threshold. Saffron, which did not produce edema, demonstrated no measurable teratogenic index. Collectively, the toxicity parameters indicate that safflower exhibited the greatest lethality based on its lower LC₅₀, whereas, among the colorants for which TI values were determined, tartrazine exhibited the comparatively higher TI, although its value remained below the classical TI > 1 threshold. Saffron exhibited the lowest toxicity across all evaluated endpoints.

**Table 1:** LC₅₀, EC₅₀, and teratogenic index (TI) values of zebrafish (*Danio rerio*) embryos exposed to saffron extract, tartrazine, and safflower extract at 96 h post-fertilization (hpf). LC₅₀ values represent the concentrations causing 50% embryo mortality, whereas EC₅₀ values indicate the concentrations producing 50% developmental abnormalities for the evaluated teratogenic endpoints. The teratogenic index (TI) was calculated as the ratio of LC₅₀ to EC₅₀ and used to estimate developmental toxicity relative to embryo lethality. Lower LC₅₀ values observed in safflower-treated groups indicated greater acute embryotoxicity, while the higher TI values associated with tartrazine reflected increased teratogenic potential. No EC₅₀ value for edema formation was determined in saffron-treated embryos because no detectable morphological abnormalities were observed at the tested concentrations. (Abbreviations: EC₅₀, median effective concentration; hpf, hours post-fertilization; LC₅₀, median lethal concentration; TI, teratogenic index; CI, confidence interval.).

			Saffron	Tartrazine	Safflower
LC_50_, % (95% CI)	0.017 (0.012 – 0.021)			0.009 (0.005 – 0.012)	0.004 (0.001 – 0.007)
Individual EC_50_, % (95% CI)					
Hatching		0.487 (0.398 – 0.541)		0.035 (0.029 – 0.041)	0.031 (0.024 – 0.040)
Cardiac & Yolk sac edema			-	0.153 (0.145 – 0.159)	0.127 (0.121– 0.134)
TI			-	0.058 (0.034 – 0.075)	0.031 (0.008 – 0.052)

### 3.6 Effects of TTZ, safflower and saffron extracts on morphological assessment of zebrafish embryo

**Fig. 6:**
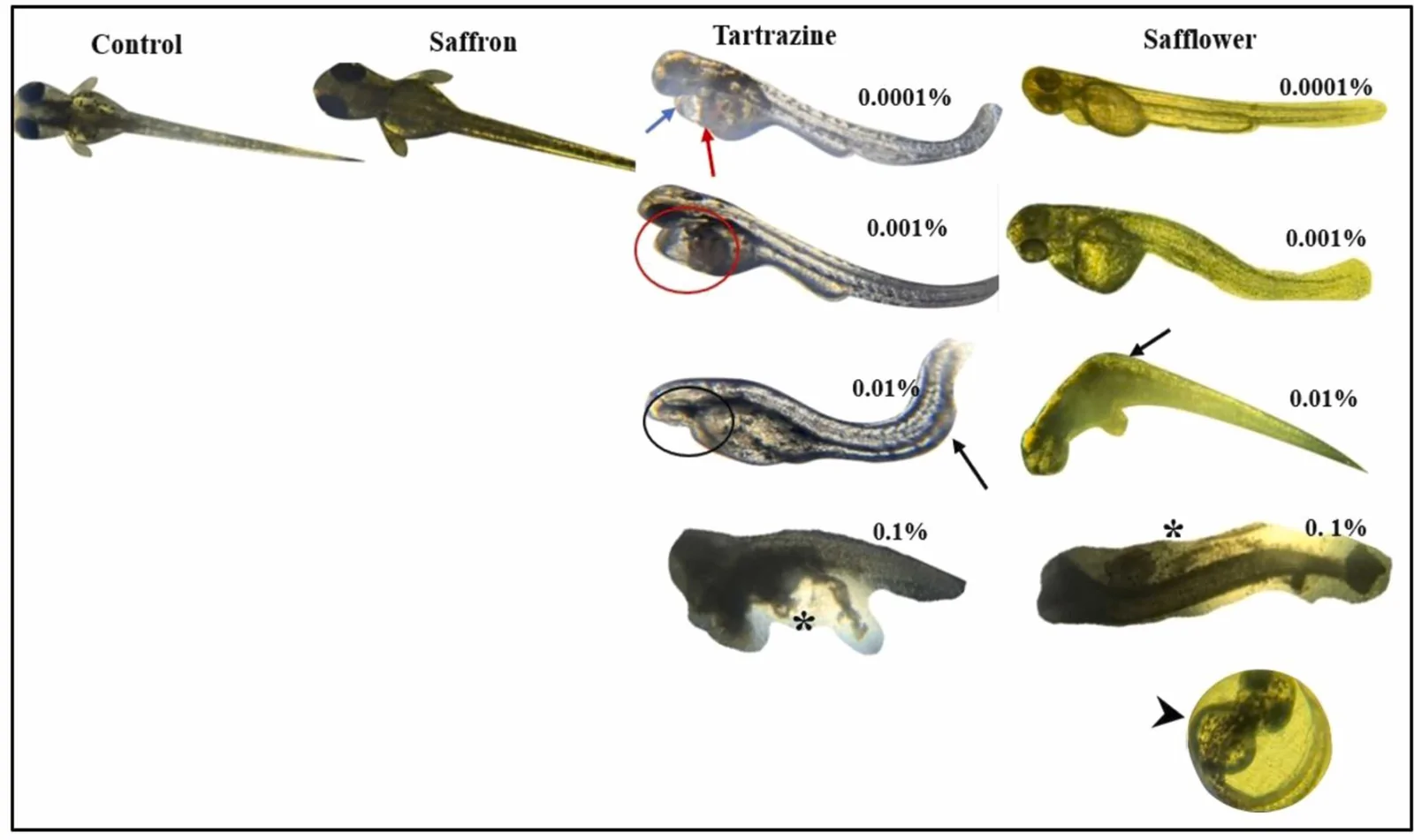
Developmental and morphological alterations observed in zebrafish *(Danio rerio)* embryos at 96 h post-fertilization (hpf) following exposure to tested food colorants. (A) Control embryos (0%) exhibited normal morphology and developmental progression. (B) Embryos exposed to saffron extract (0.0001–0.1%) showed no detectable morphological abnormalities across all tested concentrations. (C) Exposure to tartrazine (0.0001–0.1%) induced multiple concentration-dependent developmental abnormalities, including cardiac edema (red arrow), yolk sac edema (blue arrow), cardiac enlargement and severe edema formation (red circle), spinal deformities and tail curvature (black arrow), reduced or absent eye pigmentation (black circle), and embryo coagulation at 0.1% (*). (D) Safflower extract (0.0001–0.1%) caused delayed hatching, impaired embryonic growth, and reduced body length at all tested concentrations (arrowheads). These findings demonstrate differential embryotoxic and teratogenic effects among the evaluated synthetic and natural food colorants.

As described in the Morphological Scoring section (Methods), morphological abnormalities observed in each anatomical region of the embryos were documented through imaging (Fig. 6) and quantitatively scored using the six-level severity scale [42]. Quantitative analysis of morphological defects, graded on a six-level scale, revealed distinct morphological abnormalities for each treatment group. Comprehensive morphological evaluation of zebrafish embryos exposed to saffron, safflower, and tartrazine revealed pronounced differences in developmental tolerance and severity of morphological abnormalities among the three treatments. Scoring across key anatomical structures—including the tail, fins, heart, somites, pectoral fins, and facial region—showed that saffron maintained normal development at all sublethal concentrations, whereas safflower and tartrazine induced clear, dose-dependent structural deterioration (Table 2)

**Table 2:** Morphological assessment scores of zebrafish (Danio rerio) embryos exposed to saffron extract (A), safflower extract (B), and tartrazine (C) (0–1% w/v) at 96 h post-fertilization (hpf). Scores follow the six-level severity scale described in Section 2.5 (Morphological Scoring; 5 = normal, 0.5 = near-complete structural loss). Survival and hatchability are viability endpoints, reported separately in Fig. 3, Fig. 4, and are not included as morphological endpoints here. Embryos exposed to 1% were non-viable at 96 hpf and are recorded as “Dead.” The overall abnormality score is the mean of the six anatomical-region scores per group (excluding groups recorded as Dead).

**(A) Saffron extract**						
**Endpoint**	**0%**	**0.0001%**	**0.001%**	**0.01%**	**0.1%**	**1%**
Tail	5	5	5	5	5	Dead
Fins	5	5	5	5	4	Dead
Heart	5	5	5	5	5	Dead
Somites	5	5	5	5	5	Dead
Pectoral fins	5	5	5	4	5	Dead
Facial region	5	5	5	5	5	Dead
**Overall abnormality score (mean)**	**5.0**	**5.0**	**5.0**	**4.83**	**4.83**	**Dead**
**(B) Safflower extract**						
**Endpoint**	**0%**	**0.0001%**	**0.001%**	**0.01%**	**0.1%**	**1%**
Tail	5	3	2	1	0.5	Dead
Fins	5	2	2	1	0.5	Dead
Heart	5	3	2	1	1	Dead
Somites	4	3	2	1	0.5	Dead
Pectoral fins	5	2	2	1	1	Dead
Facial region	5	3	2	1	0.5	Dead
**Overall abnormality score (mean)**	**4.83**	**2.67**	**2.0**	**1.0**	**0.67**	**Dead**
**(C) Tartrazine**						
**Endpoint**	**0%**	**0.0001%**	**0.001%**	**0.01%**	**0.1%**	**1%**
Tail	5	2	3	1	0.5	Dead
Fins	5	3	3	1	0.5	Dead
Heart	5	2	2	0.5	1	Dead
Somites	5	2	2	1	1	Dead
Pectoral fins	5	2	2	1	0.5	Dead
Facial region	4	3	2	1	0.5	Dead
**Overall abnormality score (mean)**	**4.83**	**2.33**	**2.33**	**0.92**	**0.67**	**Dead**

Saffron exposure resulted in uniformly normal morphology (score = 5) up to 0.01%, with only minor, non-severe deviations (score = 4) detected in fin structures at 0.1%. No moderate or severe abnormalities occurred at viable doses, although embryos did not survive exposure at the highest concentration tested (1%). Thus, saffron elicited no substantive morphological abnormalities within the survivable concentration range. In contrast, safflower induced progressive malformations beginning at 0.0001–0.001%, with structures scoring 2–3, indicative of mild to moderate abnormalities. At 0.01%, all examined regions declined to a score of 1, demonstrating severe, multisystem disruption. Further deterioration to near-complete structural loss (score = 0.5) occurred at 0.1%, and no embryos survived at 1%, the highest concentration tested. Tartrazine produced structural abnormalities of comparable severity on the morphological scoring scale, with deficits evident at the lowest exposures. At 0.0001–0.001%, several structures scored 2–3, and by 0.01% all regions declined to a severe abnormality score of 1. At 0.1%, scores fell to 0.5–1, reflecting widespread structural collapse. Embryos failed to survive at 1%, the highest concentration tested.

Collectively, these findings demonstrate that zebrafish embryos show high developmental tolerance to saffron but marked susceptibility to safflower and tartrazine. Among the three, tartrazine and safflower produced comparatively more severe morphological abnormalities on the scoring scale, while saffron produced only minimal, isolated deviations at the highest survivable dose. It should be noted that this pattern of morphological severity is distinct from the teratogenic index (TI) values reported above: although tartrazine showed a comparatively higher TI than safflower and saffron, all three compounds' TI values remained below the classical threshold (TI > 1), and morphological severity at a given concentration should therefore be interpreted separately from the TI, which reflects the relationship between lethal and effect-associated concentrations.

### 3.7 Effects of TTZ, safflower and saffron extracts on oxidative stress (MDA Levels)

**Fig. 7:**
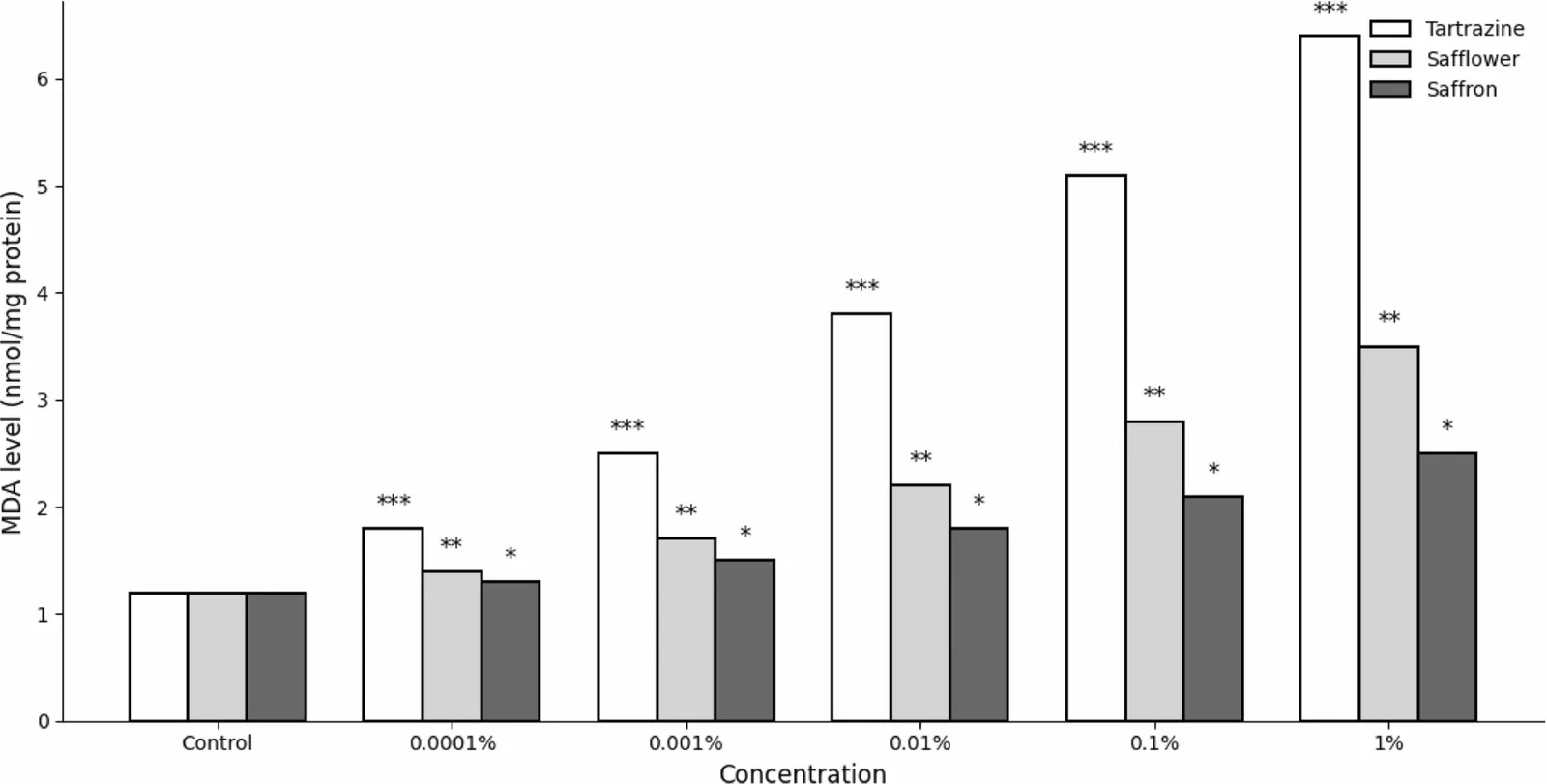
Malondialdehyde (MDA) levels in zebrafish *(Danio rerio)* larvae exposed to increasing concentrations (0.0001–1%) of saffron extract, safflower extract, and tartrazine for 96 h post-fertilization (hpf). MDA concentrations were determined as an indicator of lipid peroxidation and oxidative stress. Data are presented as Mean ± SD from three independent experiments. Significant differences compared with the untreated control group are indicated by asterisks (*P < 0.05, **P < 0.01, ***P < 0.001). Tartrazine and safflower exposure resulted in concentration-dependent increases in MDA levels, whereas saffron induced comparatively minor oxidative changes.

Malondialdehyde (MDA) levels were measured as an indicator of lipid peroxidation and oxidative stress in zebrafish embryos following exposure to tartrazine, safflower extract, and saffron extract at 96 hpf (Fig. 7). Exposure to safflower and tartrazine produced concentration-dependent increases in MDA levels relative to controls, with the highest concentrations tested (0.1 and 1%) yielding significantly elevated MDA concentrations for both substances (P < 0.05). Safflower exposure was associated with the greatest overall elevation in MDA levels, consistent with its pronounced embryotoxic and lethal effects, while tartrazine produced a comparatively smaller but still significant increase. In contrast, embryos exposed to saffron extract showed MDA levels that remained close to control values across the tested concentration range, consistent with the antioxidant properties reported for saffron constituents such as crocin, crocetin, and safranal. These findings indicate that oxidative stress, as reflected by lipid peroxidation, is a prominent feature of safflower- and tartrazine-induced embryotoxicity, whereas saffron extract did not induce a comparable oxidative response within the tested concentration range.

### 3.8 Principal component analysis findings

**Fig. 8:**
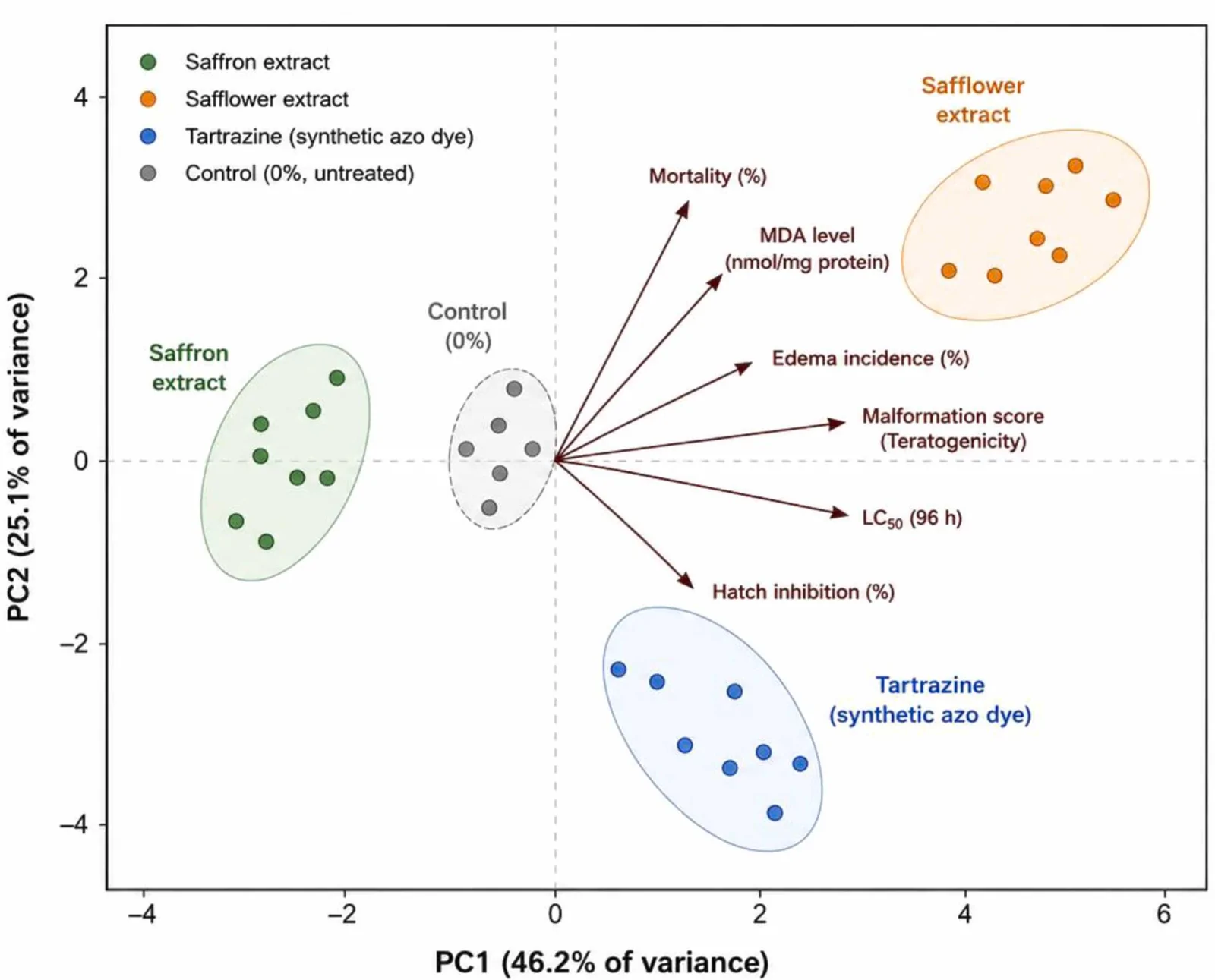
Principal component analysis (PCA) of toxicological and developmental endpoints in zebrafish embryos exposed to saffron extract, safflower extract, and tartrazine at 96 hpf. PC1 and PC2 explained 49.8% and 22.4% of the total variance, respectively. Variables associated with mortality, oxidative stress, edema, hatch inhibition, and teratogenicity contributed to the separation of treatment groups. Safflower extract clustered with embryotoxicity-related endpoints, whereas tartrazine was associated predominantly with teratogenic effects. Saffron extract clustered near the control group, indicating comparatively low toxicity.

Principal component analysis (PCA) was performed to evaluate the relationships among developmental toxicity endpoints and to compare the overall toxicological profiles of saffron extract, safflower extract, and tartrazine (Fig. 8). The first principal component (PC1) explained 49.8% of the total variance, while the second principal component (PC2) accounted for 22.4%, together representing 72.2% of the overall dataset variability. Sampling adequacy for PCA was confirmed (KMO ≥ 0.6; Bartlett’s test of sphericity, p < 0.05), and Varimax rotation was applied to aid interpretation of component loadings. The PCA biplot revealed clear separation among the tested colorants according to their biological effects. Safflower extract clustered predominantly on the positive side of PC1 and showed strong associations with mortality, edema incidence, elevated MDA levels, and reduced hatchability, indicating pronounced embryotoxicity.

In contrast, tartrazine formed a distinct cluster associated primarily with developmental abnormality endpoints, including spinal deformities and other morphological defects. Among the colorants for which TI values were determined, tartrazine exhibited a comparatively higher TI than safflower (0.058 vs. 0.031), although both values remained below the classical TI > 1 threshold. Saffron extract clustered near the control group and was associated with higher survival and hatchability rates, reflecting its comparatively low developmental toxicity. Analysis of variable loadings demonstrated that mortality, MDA level, edema incidence, and TI contributed strongly to PC1, whereas hatchability and survival were negatively associated with toxicity. Spinal deformities and developmental abnormalities contributed substantially to PC2, further distinguishing tartrazine from the other tested colorants. Overall, PCA confirmed distinct toxicological patterns among the evaluated food colorants and supported the conclusion that safflower was associated primarily with embryotoxicity and oxidative stress, whereas tartrazine clustered more closely with morphological abnormality endpoints such as spinal deformities.

Overall, PCA confirmed distinct toxicological patterns among the evaluated food colorants and supported the conclusion that safflower was associated primarily with embryotoxicity and oxidative stress, whereas tartrazine clustered more closely with morphological abnormality endpoints such as spinal deformities and edema, consistent with its comparatively higher (though sub-threshold) developmental abnormality endpoints.

## 4 Discussion

The present study comparatively evaluated the developmental toxicity and oxidative stress–inducing effects of two natural yellow food colorants, saffron and safflower, and the synthetic azo dye tartrazine using the zebrafish *(Danio rerio)* embryo model. The findings demonstrated distinct toxicological profiles among the tested colorants and revealed that toxicity was dependent not only on concentration but also on the intrinsic biological activity of each pigment source. Overall, safflower extract exhibited the greatest embryotoxicity, characterized by increased mortality, reduced hatchability, and elevated oxidative stress, among the colorants for which TI values were determined, tartrazine exhibited a comparatively higher TI than safflower (0.058 vs. 0.031), although both values remained below the classical TI > 1 threshold. In contrast, saffron extract showed comparatively low toxicity and minimal developmental disruption across most tested concentrations.

One of the most significant findings of the study was the unexpectedly high embryotoxicity associated with safflower extract. Embryos exposed to safflower demonstrated marked reductions in survival and hatching rates together with increased developmental impairment compared with both saffron and tartrazine groups. These findings indicate that natural plant-derived pigments are not inherently biologically safe and may exert substantial toxicological effects depending on their phytochemical composition and exposure concentration. Safflower petals contain diverse bioactive compounds, including flavonoids, quinochalcones, phenolic derivatives, and other secondary metabolites, some of which may interfere with embryonic signaling pathways or induce oxidative imbalance during early development. In zebrafish embryos, the chorion and rapidly differentiating tissues are highly sensitive to xenobiotic-induced oxidative and metabolic disturbances, which may explain the observed increase in mortality and developmental delay following safflower exposure. Reduced hatchability represents a sensitive indicator of developmental toxicity in zebrafish embryos and may reflect impaired embryonic growth, delayed enzymatic digestion of the chorion, or reduced spontaneous embryonic movement. In the present study, safflower exposure produced the most pronounced inhibitory effect on hatching, suggesting disruption of normal embryonic maturation processes. Delayed hatching may increase embryo vulnerability to environmental stress and may be associated with impaired cardiovascular and muscular development [43,44]. In contrast, saffron-treated embryos exhibited hatching rates comparable to controls at lower concentrations, supporting the comparatively lower developmental toxicity of saffron extract.

Although safflower exhibited the lowest 96-h LC₅₀ and therefore the greatest lethality when toxicity is integrated over the full exposure period, tartrazine produced a distinct, more acute pattern of toxicity, with rapid, near-complete mortality within 24 h at the highest concentration tested (1%). These two observations describe different aspects of the toxicity profile rather than conflicting evidence of which substance is “most toxic”: safflower’s lower 96-h LC₅₀ reflects greater potency under prolonged, low-to-moderate exposure, whereas tartrazine’s rapid effect at the highest concentration reflects greater acute, high-dose lethality. A comparatively higher TI was observed for tartrazine than for safflower (0.058 and 0.031, respectively), with both values remaining below the classical TI > 1 threshold. This finding indicates a narrower margin between the lethal and malformation-inducing concentrations of tartrazine, together with higher frequencies of cardiac edema, yolk sac edema, spinal deformities, tail curvature, and pigmentation abnormalities. These findings suggest that tartrazine exerts developmental toxicity primarily through disruption of morphogenetic and organogenic pathways rather than through direct embryonic lethality alone. Azo dyes such as tartrazine have been reported to generate reactive metabolites and oxidative intermediates capable of interfering with mitochondrial function, cellular differentiation, and embryonic signaling cascades [45,46]. The observed cardiac and yolk sac edema may reflect impaired osmoregulatory and cardiovascular development, whereas spinal curvature and tail malformations indicate disruption of musculoskeletal morphogenesis during somitogenesis and axial patterning [47,48].

**Fig. 9:**
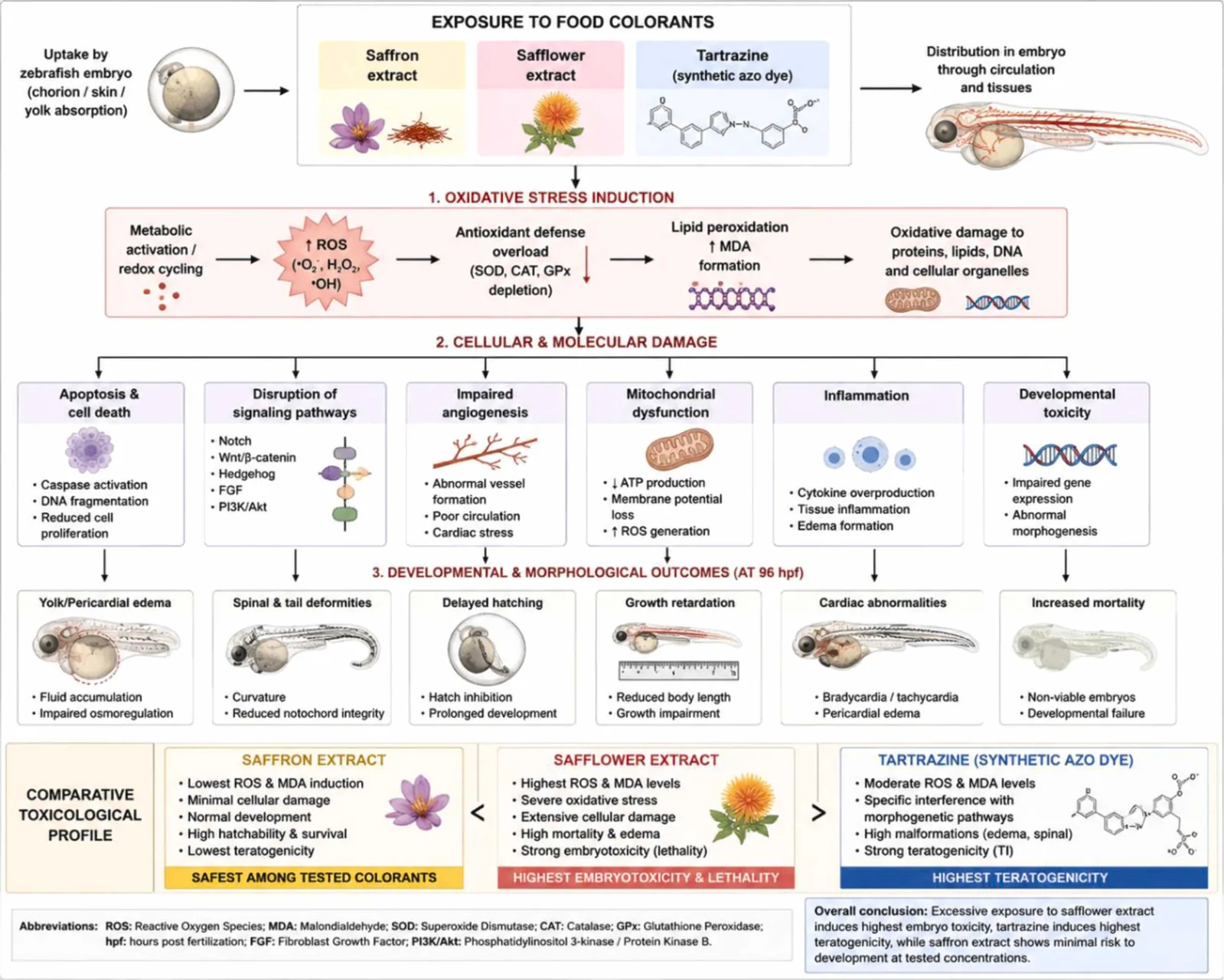
Proposed mechanism underlying developmental toxicity induced by yellow food colorants in zebrafish (*Danio rerio*) embryos. Exposure to tartrazine and safflower extract promotes reactive oxygen species (ROS) generation, leading to oxidative stress and lipid peroxidation, as evidenced by increased malondialdehyde (MDA) production. Oxidative damage may subsequently disrupt cellular homeostasis, mitochondrial function, and developmental signaling pathways, resulting in edema formation, spinal deformities, delayed hatching, growth impairment, and increased embryonic mortality. Tartrazine was associated predominantly with teratogenic effects, whereas safflower exhibited greater embryotoxicity and lethality. Saffron extract demonstrated comparatively low toxicity and minimal oxidative damage. The proposed pathways are based on the findings of the present study together with previously reported mechanisms of food colorant-induced developmental toxicity.

The proposed mechanistic pathways underlying food colorant–induced developmental toxicity are summarized schematically in Fig. 9. The observed increase in oxidative stress biomarkers together with morphological abnormalities suggests that excessive reactive oxygen species (ROS) generation and lipid peroxidation may play central roles in the embryotoxic and developmental morphological abnormalities of the tested colorants [49]. MDA is a major end-product of lipid peroxidation and serves as a reliable biomarker of oxidative membrane damage. Elevated MDA concentrations in safflower- and tartrazine-treated groups indicate increased reactive oxygen species (ROS) generation and oxidative injury during embryonic development. Excessive ROS production may damage membrane lipids, proteins, and nucleic acids, leading to altered cellular homeostasis, apoptosis, impaired organogenesis, and developmental defects. The stronger association between safflower exposure and MDA elevation suggests that oxidative stress may be a principal mechanism underlying the high embryotoxicity observed in this group. Conversely, the relatively lower MDA levels detected in saffron-treated embryos may be attributable to the antioxidant properties of saffron constituents such as crocin, crocetin, and safranal, which have been reported to possess free radical scavenging activity [50,51].

The differential toxicological profiles observed between safflower and tartrazine are particularly noteworthy. While safflower exposure was associated mainly with lethality and impaired hatching, tartrazine exposure was more strongly linked to structural malformations and morphological abnormalities. This distinction suggests that the tested colorants may act through partially different mechanisms of developmental toxicity. Safflower-induced toxicity appears to be associated predominantly with generalized cellular and oxidative damage, whereas tartrazine may interfere more directly with developmental signaling pathways governing embryonic patterning and organ formation. Such mechanistic differences are supported by the PCA analysis, which revealed distinct clustering patterns among the tested compounds.

The PCA findings provided integrative multivariate confirmation of the observed toxicological trends. Safflower extract clustered closely with mortality, oxidative stress, edema formation, and hatch inhibition, indicating a strong association with embryotoxic endpoints. In contrast, tartrazine clustered predominantly with malformation-related variables, including spinal deformities and developmental abnormalities, suggesting a stronger contribution to developmental dysregulation rather than generalized lethality. Saffron extract clustered near the control group and favorable developmental parameters, further supporting its comparatively low toxicity. The PCA loading vectors also demonstrated that oxidative stress markers and mortality contributed strongly to the principal variance components, reinforcing the central role of oxidative injury in food colorant–induced embryotoxicity.

Importantly, the findings of the present study challenge the widespread assumption that natural food colorants are universally safer than synthetic additives. Although consumers often perceive plant-derived colorants as harmless alternatives to synthetic dyes, the present results demonstrate that natural origin alone does not guarantee biological safety. Safflower extract exhibited greater embryotoxicity than tartrazine in several endpoints, including lethality and hatch inhibition. This observation highlights the necessity of rigorous toxicological evaluation for both natural and synthetic food additives. Toxicity should therefore be assessed based on biological effects, exposure concentration, chemical composition, and mechanistic evidence rather than solely on whether a compound is classified as “natural” or “synthetic.”

The zebrafish embryo model provides several advantages for developmental toxicology research, including rapid external development, optical transparency, genetic homology with humans, and sensitivity to environmental toxicants. Many molecular pathways involved in oxidative stress responses, cardiovascular development, neurodevelopment, and organogenesis are highly conserved between zebrafish and mammals [52,53]. Consequently, zebrafish embryotoxicity studies are considered valuable screening tools for identifying compounds with potential developmental hazards relevant to human health. Nevertheless, direct extrapolation of zebrafish toxicity data to humans should be approached cautiously due to interspecies differences in metabolism, toxicokinetics, absorption, and exposure conditions. Additional mammalian studies and mechanistic investigations are therefore necessary to confirm the translational relevance of the present findings.

Several limitations should also be acknowledged. The study focused primarily on acute embryotoxicity and early developmental endpoints up to 96 hpf, whereas long-term behavioral, reproductive, and neurodevelopmental effects were not assessed. Furthermore, the complex phytochemical composition of safflower and saffron extracts was not chemically characterized in detail, and therefore the specific constituents responsible for the observed toxic effects remain unclear. In addition, safflower and saffron extracts were prepared using different initial mixing-water temperatures, selected to mirror typical culinary practice for each material; although both extracts subsequently underwent an identical hydrothermal extraction step, this initial temperature difference is a methodological variable that could itself influence the resulting phytochemical profile and should be controlled for (e.g., using matched extraction temperatures) in future comparative studies. Future studies involving chromatographic characterization, gene expression analysis, antioxidant enzyme profiling, and long-term developmental assessment would provide deeper mechanistic insight into the biological effects of these food colorants.

In conclusion, the present study demonstrated that both natural and synthetic yellow food colorants can induce developmental toxicity and oxidative stress in zebrafish embryos in a concentration-dependent manner. Safflower extract was associated with greater mortality and more pronounced hatch inhibition. The TI value for tartrazine was comparatively higher than that for safflower (0.058 vs. 0.031); however, both values remained below the classical TI > 1 threshold. Saffron extract was associated with comparatively limited developmental and morphological effects under the tested conditions. These findings emphasize the importance of comprehensive toxicological evaluation of food additives regardless of their natural or synthetic origin and highlight oxidative stress as a central mechanism contributing to food colorant–induced developmental toxicity.

## 5 Recommendations

Future studies should incorporate transcriptomic, proteomic, and metabolomic profiling to elucidate disrupted pathways and verify the formation of reactive intermediates. Assessments of mitochondrial function, and apoptosis will clarify mechanistic links to developmental abnormalities. Long-term evaluations, including behavioral and reproductive endpoints, are needed to determine whether early embryonic disturbances translate to persistent deficits.

## CRediT authorship contribution statement

**Mahdieh Kiannezhad:** Writing – original draft, Investigation, Data curation. **Jalal Hassan:** Visualization, Supervision, Project administration, Methodology. **Mohammad Kazem Koohi:** Methodology. **Amir Moghaddam Jafari:** Writing – review & editing, Writing – original draft, Supervision, Project administration, Conceptualization.

## Funding

This work was supported by the veterinary faculty and the Vice-Chancellor of Research, Ferdowsi University of Mashhad, Mashhad, Iran.

## Declaration of Competing Interest

The authors declare that they have no known competing financial interests or personal relationships that could have appeared to influence the work reported in this paper.

## Data Availability

Data will be made available on request.
